# Parallel Evolution of Enhanced Biofilm Formation and Phage-Resistance in *Pseudomonas aeruginosa* during Adaptation Process in Spatially Heterogeneous Environments

**DOI:** 10.3390/microorganisms9030569

**Published:** 2021-03-10

**Authors:** Kyosuke Yamamoto, Hiroyuki Kusada, Yoichi Kamagata, Hideyuki Tamaki

**Affiliations:** 1 Bioproduction Research Institute, National Institute of Advanced Industrial Science and Technology (AIST), Sapporo 0628517, Hokkaido, Japan; 2 Bioproduction Research Institute, AIST, Tsukuba 3058566, Ibaraki, Japan; kusada-hiroyuki@aist.go.jp (H.K.); y.kamagata@aist.go.jp (Y.K.); 3 Faculty of Life and Environmental Sciences, University of Tsukuba, Tsukuba 3058577, Ibaraki, Japan; 4 Biotechnology Research Center, University of Tokyo, Bunkyo-ku, Tokyo 1138657, Japan

**Keywords:** *Pseudomonas aeruginosa*, evolution, spatial heterogeneity, interspecies interaction, biofilm, *Staphylococcus aureus*

## Abstract

An opportunistic pathogen *Pseudomonas aeruginosa* has a versatile phenotype and high evolutionary potential to adapt to various natural habitats. As the organism normally lives in spatially heterogeneous and polymicrobial environments from open fields to the inside of hosts, adaptation to abiotic (spatial heterogeneity) and biotic factors (interspecies interactions) is a key process to proliferate. However, our knowledge about the adaptation process of *P. aeruginosa* in spatially heterogeneous environments associated with other species is limited. We show herein that the evolutionary dynamics of *P. aeruginosa* PAO1 in spatially heterogeneous environments with *Staphylococcus aureus* known to coexist in vivo is dictated by two distinct core evolutionary trajectories: (i) the increase of biofilm formation and (ii) the resistance to infection by a filamentous phage which is retained in the PAO1 genome. Hyperbiofilm and/or pili-deficient phage-resistant variants were frequently selected in the laboratory evolution experiment, indicating that these are key adaptive traits under spatially structured conditions. On the other hand, the presence of *S. aureus* had only a marginal effect on the emergence and maintenance of these variants. These results show key adaptive traits of *P. aeruginosa* and indicate the strong selection pressure conferred by spatial heterogeneity, which might overwhelm the effect of interspecies interactions.

## 1. Introduction

An opportunistic pathogen *Pseudomonas aeruginosa* has a versatile phenotype that enables the bacterium to adapt to a wide variety of natural habitats from open fields to the inside of hosts. This versatility is underpinned by diverse metabolic pathways utilizing various growth substrates [1], high resistance to a wide range of stress agents [2], and switching between planktonic and sessile growth modes by motility and formation of multicellular structure known as biofilms [3,4]. In addition to such phenotypic plasticity *per se*, the evolutionary potential of *P. aeruginosa* can strengthen and broaden the versatile abilities described above and also contribute to its adaptation and proliferation in diverse habitats, making the pathogen recalcitrant and troublesome in clinical settings [5,6].

Most natural microbial habitats are spatially heterogeneous, and a preferred way to adapt to such structured environments is the development of a sessile biofilm growth mode. *P. aeruginosa* can form biofilms at various interfaces and the biofilm formation ability is often enhanced during the adaptation process to their habitat; strains showing increased production of biofilm matrix (e.g., extracellular polysaccharides), known as hyperbiofilm variants, are frequently isolated from in vivo clinical settings [7] and rapidly emerge in an in vitro single-batch biofilm culture within a couple of days [8]. However, evolutionary constraints for emergence and maintenance of a hyperbiofilm phenotype in spatiotemporally heterogeneous habitats (e.g., soil microstructure and the inside of hosts) still remain elusive. In particular, a fitness cost of hyperbiofilm phenotype has not been evaluated well, and it is very unclear if there are any fitness trade-offs between the enhanced biofilm matrix production and other phenotypes. For instance, overproduction of biofilm matrix would be beneficial for local competitiveness but can reduce migration efficiency due to limiting dispersal [9,10] (competition-colonization trade-off). Therefore, a hyperbiofilm phenotype may have a maladaptive effect by affecting other traits such as motility and attachment, which are crucial traits in the course of repeated biofilm-planktonic lifecycles (e.g., initial migration to the surface and cell dispersal from matured biofilms), and some compensative mutations might be needed for the maintenance of hyperbiofilm phenotype in repeated biofilm-planktonic lifecycles, not in single-batch biofilm cultures. Besides, clinical isolates, mainly isolated from the lungs of cystic fibrosis (CF) patients, do not always show an exclusive biofilm formation [6], indicating the existence of distinct adaptive traits which can confer fitness advantages from hyperbiofilm phenotype. To understand the successful adaptation process of *P. aeruginosa* to heterogeneous environments, it is crucial to evaluate the emergence, proliferation, and maintenance or extinction of various evolved strains including hyperbiofilm variants in the course of the repeated biofilm-planktonic lifecycles. 

In addition to abiotic factors such as spatial heterogeneity, biotic factors are also driving forces for evolution [11]. In the case of pathogens, the interaction with host immune systems has been intensively investigated as determinants for pathogen evolution due to its strong selective pressures [12,13]. In addition to such host–pathogen interaction, interspecies interactions among bacteria are also key factors [14,15,16]. *P. aeruginosa* is known to interact with other bacteria in infected sites [17]. In particular, *Staphylococcus aureus*, which is a well-known notorious pathogen, frequently coexists with *P. aeruginosa* in their host and thereby has been considered as an important interaction partner for *P. aeruginosa* in vivo [18,19,20]. Most of the previous studies have highlighted the competitive interaction between them represented by the interference of *S. aureus* by *P. aeruginosa* [14,21]. Conversely, it has been reported that *S. aureus* modulates *P. aeruginosa* transcription profiles [22] and physiology [23,24,25,26], provoking that *S. aureus* can affect the evolutionary dynamics of *P. aeruginosa*. Indeed, a few works indicated that interactions with *S. aureus* can alter the evolutionary trajectories of *P. aeruginosa* [27]. However, the experimental setting implemented there was rather artificial as a large amount of *S. aureus* cells were amended periodically to the *P. aeruginosa*-*S. aureus* coculture system during evolution experiment. Therefore, evolutionary consequence of reciprocal interactions along with spontaneous population dynamics originated from initial populations has not been evaluated.

Thus, whereas a number of studies focusing on lifestyles of *P. aeruginosa* in structured environments (e.g., biofilm formation) and interactions with *S. aureus* (e.g., interference) have been conducted as cited above, the evolutionary consequence of such ecological processes has rarely been experimentally investigated. In this study, we evaluated the adaptive divergent process of *P. aeruginosa* in spatially heterogeneous environments, focusing on not only the emergence but also the maintenance of evolved variants during a prolonged culture period with periodic habitat disturbances. Furthermore, the effect of interspecies interaction with *S. aureus* on the evolutionary process of *P. aeruginosa* was also evaluated in terms of spontaneous eco-evolutionary feedbacks where reciprocal interaction was maintained between original populations without periodic addition of cells from outside. *P. aeruginosa* was experimentally evolved under static culture conditions with a serial dilution regime in the absence or the presence of competitor species, *S. aureus*. We hypothesize that certain kinds of adaptive phenotypes would emerge and be selected during the passages of cultivation, which may be key phenotypes for thriving in various spatially-heterogenous environments, and that patterns of evolution of such key phenotypes (styles of phenotypic change, rate of persistence, frequency of emergence, etc.) would be changed by interactions with *S. aureus*. The evolutionary trajectory of *P. aeruginosa* population was assessed by the emergence and temporal changes in abundances of colony morphology variants (CMVs) because colony morphology is known to change accompanied with phenotypic innovation including hyperbiofilm phenotype. Phenotypic and genomic features of CMVs underpinning the observed evolutionary dynamics were investigated by physiological analyses and whole-genome resequencing, respectively.

## 2. Materials and Methods

### 2.1. Strain and Medium

The bacterial strains used in this study were *P. aeruginosa* PAO1 (PAO1), *S. aureus* Newman (SN) [28], and *S. aureus* Mu50 (SM) [29]. SN and SM were chosen as representatives of methicillin-sensitive and -resistant *S. aureus* strain, respectively, which have distinct phenotypic characteristics and, therefore, are expected to show distinct effects on ecology and evolution of *P. aeruginosa*. These strains were used as ancestral strains of *P. aeruginosa* and *S. aureus* and routinely grown in Luria–Bertani (LB) medium at 37 °C under static or shaking conditions. All static cultures were performed in short test tubes (Pyrex glass, inner diameter 13 mm, length 85 mm) with 3 mL of medium. Cultures were inoculated with 1% (*v/v*) of preculture (overnight culture).

### 2.2. Evolution Experiment

The evolution experiment was performed in a daily serial transfer regime. Three culture series were constructed and subjected to serial transfer: a pure culture of PAO1 (P), a coculture of PAO1 and SN (PN), and a coculture of PAO1 and SM (PM). Each culture was inoculated with 1% (*v/v*) of preculture into 3 mL of LB medium in a short test tube with a loose cap and incubated at 37 °C under static conditions. After 24 h cultivation, each culture was homogenized by vortexing for 2–3 min, and then 1% (*v/v*) of culture was transferred into fresh LB medium to give the next round. Population size and structure of PAO1 and *S. aureus* in each culture were evaluated by enumeration of viable cell numbers in the homogenized culture as described below. Each culture sample was stored at −80 °C after adding glycerol at the final concentration of 16% (*v/v*) and revived for investigating the population structure when needed. Two independent experiments were performed, and each experiment had 5 replicates for each culture series, making 10 lines per culture series in total.

### 2.3. Viable Cell Number and Colony Discrimination

Viable cell number was estimated by colony-forming unit (CFU) counting. Dilution series were made from the homogenized culture with sterilized phosphate-buffered saline (PBS) and spread onto tryptic soy agar plates (tryptic soy broth [BD, Franklin Lakes, NJ, USA] with 1.5% [*w/v*] agar) for PAO1 and mannitol salt agar (MSA [BD]) plates for *S. aureus*, then incubated for 1–2 days at 37 °C or 30 °C. Discrimination and classification of CMVs were performed by visual observation, and colonies of ancestral strains and CMVs were separately counted. 

### 2.4. Isolation and Phenotypic Characterization of CMVs

Representative colonies of each CMVs were picked up from the plates and purified by repeated streaking and picking up of colony (at least two times). Purified CMVs were propagated in LB medium overnight and stored at −80 °C with glycerol at a final concentration of 16% (*v/v*). 

Twitching motility was evaluated by the size of twitching zone. Strain was revived by direct streaking of frozen stocks onto LB agar and overnight incubation. Colonies of each strain were picked and stub-inoculated by toothpick onto the bottom of LB agar (1.0% [*w/v*] agar) in the petri dish, and incubated at 37 °C for 48 h, followed by measurement of the twitching zone. 

Biofilm formation was evaluated by the crystal violet staining method [30]. Each culture was inoculated with 1% (*v/v*) of preculture into 100 μL of LB medium in a well of 96-well microtiter plate (OD_600_ = approximately 0.02–0.05) and incubated at 37 °C under static conditions. After 24 h cultivation, biofilm in each well was stained with 20 μL of 0.1% (*w/v*) crystal violet solution (Wako, Osaka, Japan) for 10 min at room temperature. Residual dye and unattached fraction were discarded, and then well was washed with PBS three times. After dry-up at room temperature for 15 min, 100 μL of 100% (*v/v*) ethanol was added into the well to elute dye-stained biofilm fraction. Elution was performed for 15 min with mild shaking, followed by OD_600_ measurement with a microplate reader (Thermo Labsystems Multiskan Spectrum UV/visible Microplate Reader, Thermo Labsystems, Waltham, MA USA).

Susceptibility to temperate phage was evaluated by spot test with double-layer agar plates. Phage particles were collected from Sm-dominating culture by filtration of broth using 0.22 μm pore size filter and then amplified by superinfection with PAO1 cells and lysis using a double layer agar method described below. Phage solution was prepared by dispersing phage particles within top agar layer into PBS poured onto top agar layer. Top agar was prepared by mixing 5 mL molten LB agar (0.5% [*w/v*] agar) with 100 μL of overnight culture of each strain and poured onto 15 mL base LB agar (1.5% [*w/v*] agar) in a petri dish. After top agar was solubilized, 10 μL of dilution series of the phage solution was spotted onto top agar, and then incubated for 24 h at 37 °C. Susceptibility was evaluated by the formation of a clear zone at the spotted area on the top agar.

### 2.5. Genome Resequencing Analysis

For resequencing of evolved isolates, genomic DNA (gDNA) of ancestral PAO1 and each CMV were extracted from overnight cultures using illustra bacteria genomicPrep Mini Spin Kit (GE healthcare Life Sciences, Pittsburgh, PA USA) according to manufacturer’s instruction. The gDNA was sequenced with an Illumina Miseq (Illumina, San Diego, CA USA) with V2 PE250 reagents kit which generated paired-end reads of 250 nucleotides (nt) by Otogenetics Corporation (Norcross, GA USA). Obtained read numbers par sample ranged from 923,484 to 3,695,416 (mean value 2,459,455), representing genome coverages ranging from 37x to 148x (mean value 98x). Read mapping and variant detection were performed by CLC Genomics Workbench ver.8.0 (CLC bio, Aathus, Denmark). Obtained reads were mapped into the reference PAO1 genome, and nucleotide variants were detected by the quality-based method. The nucleotide variation found in over 97% of covered reads (average 99.8%) were defined as sequence variants in the genome of the evolved isolate. Detected variants were confirmed by specific PCR and Sanger sequencing.

### 2.6. Analysis of Diversity and Evolutionary Trajectories

The evolutionary trajectory of *P. aeruginosa* population was evaluated by a temporal transition of CMV composition. Population structure of all samples at all time points based on proportion of CMVs were pairwise compared to generate a resemblance matrix based on Bray-Curtis similarity. The resemblance matrix was analyzed by non-metric multidimensional scaling (nMDS) and plotted onto 2D plane using PRIMER 6 (PRIMER-E, Auckland, New Zealand). The trajectory of each culture line was visualized by connecting all time points.

## 3. Results

### 3.1. Emergence of Colony Morphology Variants (CMVs) of P. aeruginosa PAO1

**Figure 1:**
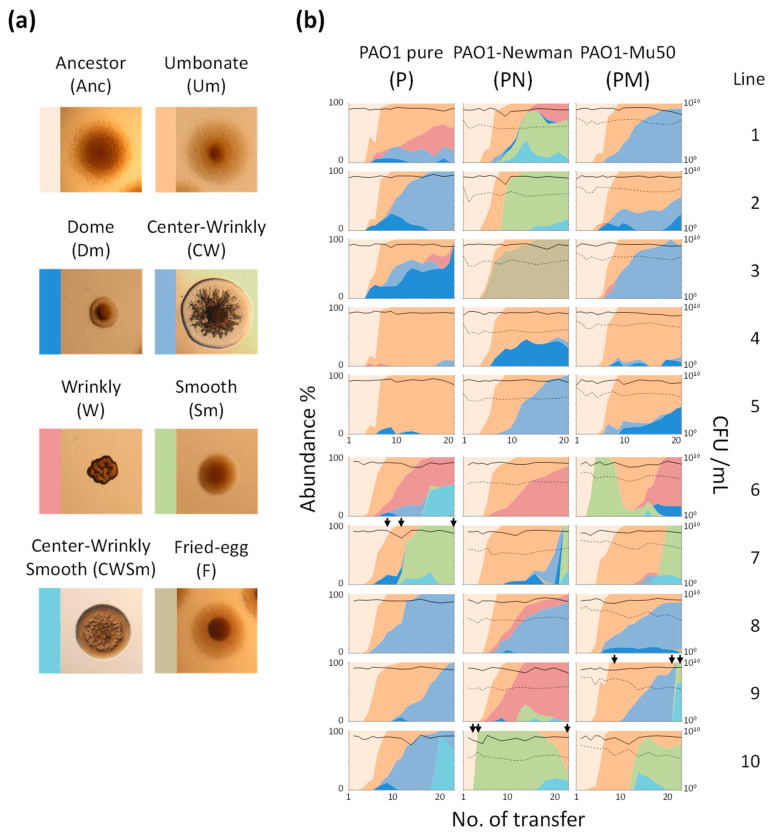
Evolutionary and population dynamics of *Pseudomonas aeruginosa*. (**a**) Typical colony morphologies of major colony morphology variants (CMVs). (**b**) Transition of population size and abundance of CMVs. Area plots indicate proportion of each CMV, and colors correspond to CMVs shown in (a). Solid lines and dashed lines indicate viable cell number (CFU/mL) of PAO1 and each *S. aureus* strain, respectively. Missing data point was interpolated by connecting the value at before and after gap to create smooth area plots. Complete data sets were provided in Supplementary Figure S1. Total numbers of transfer were 21 and 23 for Line 1 to 5 and Line 6 to 10, respectively. Black arrows indicate time points at which the concentration of temperate phage in the culture supernatant was quantified.

Three culture series were constructed in the evolution experiment and subjected to serial transfer: a pure culture of *P. aeruginosa* PAO1 (P), a coculture of PAO1 and *S. aureus* strain Newman (PN), and a coculture of PAO1 and *S. aureus* strain Mu50 (PM). In the course of the evolution experiment, PAO1 maintained a high viable cell density (approximately 10^8^–10^9^ CFU/mL) irrespective of the existence of *S. aureus* strain Newman (SN) and strain Mu50 (SM) (Figure 1b). On the other hand, population sizes of SN and SM (approximately 10^6^–10^7^ CFU/mL) were sharply dropped at the beginning of the subculture and, thereafter, constantly smaller than that of PAO1 most probably due to growth interference by PAO1 as previously described [18]. Nevertheless, *S. aureus* strains did not go extinct, and they constantly coexisted and interacted with PAO1 in the course of the subculture.

PAO1 diverged into several CMVs during the evolution experiment (Figure 1). A dominant CMV especially in early time points was an umbonate-form variant (Um). The Um emergence was a common event in all culture series and was observed within 3rd to 5th rounds of transfer in most lines (Figure 1b). Although the Um variants kept dominant after their emergence in some cases (e.g., lines P-4, P-5, and PM-4), wrinkly-type CMVs (W, CW, and F) emerged after or parallel to a transition from ancestor to the Um dominance and gradually predominated in many culture lines. A dome-shaped CMV (Dm) also frequently emerged but it did not overwhelm other variants with one exception (P-3). 

Smooth-type CMVs (Sm and CWSm) were detected in some culture lines at various timings. Notably, the Sm variants rapidly predominated after its emergence in most cases, indicating their large fitness advantage under the present conditions. Thus, all PAO1 populations diverged from clonal ancestral population into the mixture of various CMVs during successive cultivation in spatially-structured environments, and the transition patterns varied by populations. It is noteworthy that no wrinkly-type CMVs and few smooth-type CMVs were detected in the course of the evolution experiment performed under shaking conditions (no spatial structure) (Supplementary Figure S2), showing that the evolutionary dynamics described above was strongly attributed to spatially-heterogeneous environments.

### 3.2. Genomic and Phenotypic Characterization of CMVs

To clarify the genetic backgrounds for the variation of colony morphologies and obtain insights into ecological process affecting the evolutionary dynamics of PAO1, a whole-genome resequencing analysis was performed in 11 representative CMVs (Um, Dm, F, CW, W, and Sm) isolated from line 1 to 5 (Table 1). 

**Figure 2:**
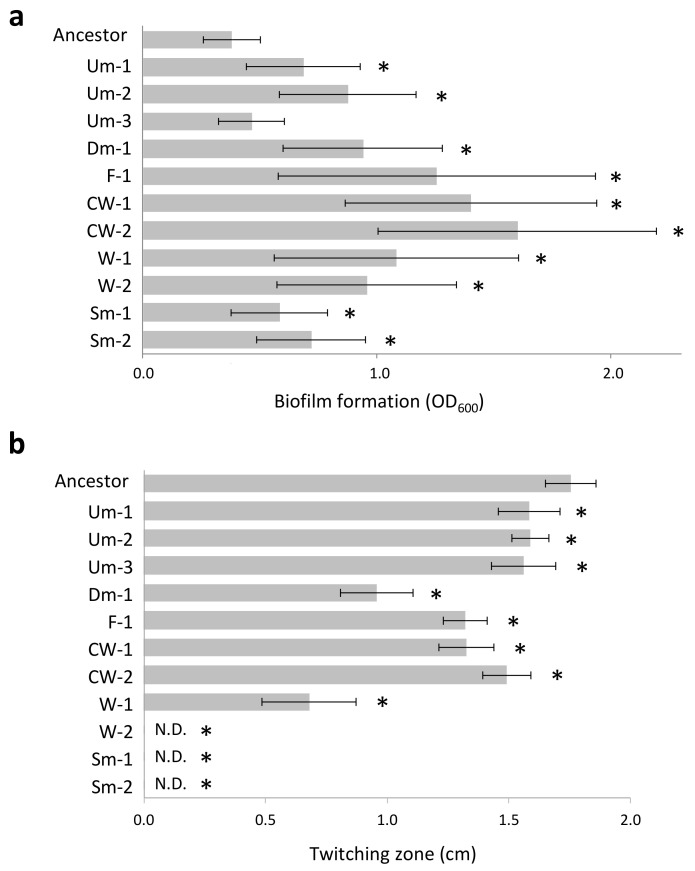
Phenotypic features of CMVs. (**a**) Biofilm formation (24 h). Values are expressed as means for three independent experiments (*n* = 6). Error bars indicate SD. Asterisks indicate statistically significant difference to Ancestor (student’s *t*-test, *p* < 0.01). (**b**) Twitching motility (48 h). Values are expressed as means for three independent experiments (*n* = 3–5). Error bars indicate SD. Asterisks indicate statistically significant difference to Ancestor (student’s *t*-test, *p* < 0.01). N.D., Not detected.

The genomes of the wrinkly CMVs and the Dm variant harbored mutations (indels and substitutions) in coding sequences of genes related to c-di-GMP metabolism (*wspA*, *wspF*, *morA*, *yfiB*, and PA0171). It has been known that wrinkly colony type emerges during biofilm development processes and shows a hyperbiofilm phenotype due to an overproduction of exopolysaccharides [8]. In accordance with this, the CMVs harboring these mutations exhibited enhanced biofilm formation (Figure 2a), and increased pellicle formation at the air–liquid interface was observed in the culture lines predominated by these CMVs (data not shown). Another group of genes harboring mutations was related to production and/or regulation of type IV pili (*pilA* and *pilJ*) which is a cell surface structure involving motility and attachment. These mutations were mainly found in the genomes of Sm variants which exhibited complete defection of twitching motility (Figure 2b). The Um variants also possessed genotypes that resemble those of the Sm variants or had no mutation in its genome (Um-3), but the Um variants exhibited twitching motility compatible with the ancestral PAO1 strain (Figure 2b). The results indicate that the c-di-GMP metabolism and the regulation of type IV pili are two important mutational targets in the present adaptation process of PAO1.

### 3.3. Selection of Sm Variants by Phage Lysis

**Figure 3:**
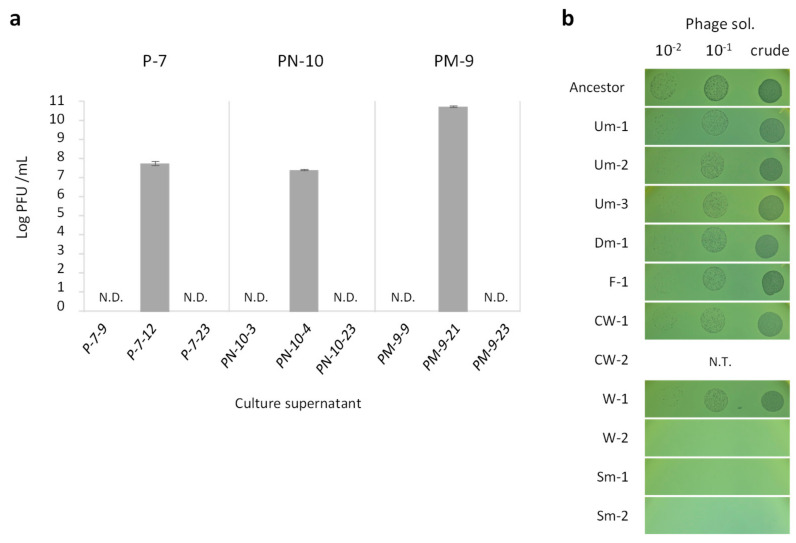
Detection of temperate phage and phage resistance of CMVs. (**a**) Bacteriophage titer was expressed as plaque forming unit (PFU)/mL in the culture supernatant indicated (see Figure 1). Values are expressed as means for three independent experiments (*n* = 3). Error bars indicate SD. (**b**) Susceptibility of CMVs to temperate phage. Phage solution and its dilution series were spotted onto a lawn of strains indicated. Susceptibility of CW-2 was not evaluated by spot test due to its exclusive aggregation phenotype preventing the formation of lawn in top agar.

Although the proliferation of wrinkly type CMVs can be explained by the fitness advantage conferred by increased biofilm formation [31], it was still unclear if the pili deficiency found in smooth type CMVs (Sm variants) provides fitness advantages to PAO1. The competition assay indeed showed that the loss of type IV pili itself did not confer any fitness advantage to PAO1 (data not shown). We then assumed that type IV pili deficiency would be associated with resistance to phage lysis since the pili are a recognition target of the temperate phages [32]. *P. aeruginosa* possesses the Pf4 prophage in its genome, which is induced into a lytic cycle during biofilm growth [33], and the released phage particles can superinfect and lyse sibling *P. aeruginosa* cells. In fact, a decrease of viable cell numbers was observed prior to predominance of Sm in most cases (e.g., P-7, PN-1, and PN-2 in Figure 1b). As expected, a considerable number of active phages were detected in the supernatant of Sm-proliferating cultures at the timing of sharp increase of Sm abundance (Figure 3a), and PCR and sequencing analyses showed the existence of the replicative-form DNA of Pf4 in these culture supernatants (Supplementary Figure S3). In addition, the Sm variants exhibited higher resistance to phage infection comparing with those of the ancestral PAO1 and other CMVs which have intact type IV pili (Figure 3b). Taken together, our results indicate that the cell lysis by temperate phages occurred in the biofilm population at various timings in the course of culture, and then the phage-resistant Sm variants were selected and proliferated.

### 3.4. Evolutionary Trajectory of PAO1 Population

**Figure 4:**
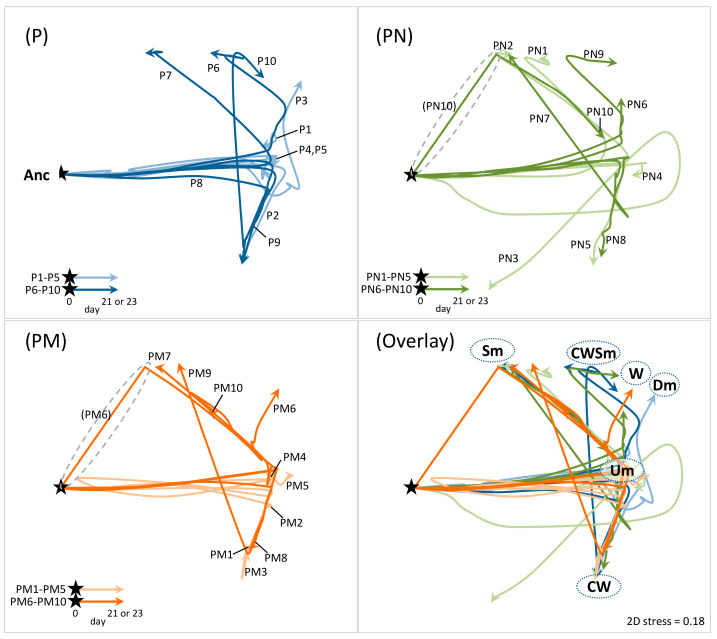
Temporal trajectories of *P. aeruginosa* population structure. 2D non-metric multidimensional scaling (nMDS) plot was created based on community resemblance (Bray–Curtis similarity) matrix generated from time course data of abundance of ancestor and CMVs in each culture shown in Figure 1b (see Material and Methods). Each arrow line corresponds to each culture line in Figure 1b (ten lines each for the PAO1 pure culture [P], the coculture with *S. aureus* Newman [PN], and the coculture with *S. aureus* Mu50 [PM]). Filled stars indicate starting point (time zero; 100% ancestor), and trajectories of arrow line represent the transition of population structure. The more positions in the 2D pane are closed to each other, the more population structures are similar. Name of CMVs on the plot indicates a position of the culture whose population was almost fully predominated by indicated CMVs. Grey dashed circles highlight trajectories observed specifically in the coculture with *S. aureus*.

To compare the adaptation processes of PAO1 population among the culture lines, the temporal transition of population structure in each culture line was visualized on 2D non-metric multidimensional scaling (nMDS) plots based on Bray–Curtis similarity matrix, which was created by all-to-all pairwise comparison of variant composition among all culture lines at all sampling points (Figure 4; Each arrow line corresponds to each culture line in Figure 1b). The Um variants proliferated first in most culture lines, and then Um dominance was kept in some lines (e.g., P-4, P-5, and PM-4) and the hyperbiofilm variants CW (e.g., P-2, P-8, P-9, PN-5, PN-8, PM-1, PM-3, and PM-8), W (e.g., PN-6, PN-9, and PM-6), and Dm (e.g., P-3) or the smooth-variants Sm (e.g., P-7, PN-2, PN-7, and PM-7) and CWSm (e.g., P-10) finally predominated in other lines. Such transition pattern was commonly observed in both PAO1 pure cultures (P) and two cocultures (PN and PM). A unique transition pattern in the coculture series was observed in the limited lines: the early emergence and the rapid persistence of Sm variants without going through the Um proliferation (PN10 and PM6; highlighted with dashed circle in Figure 4).

## 4. Discussion

Adaptive divergence is a complex process governed by biotic and abiotic ecological and evolutionary factors and has recently attracted much attention in the context of eco-evolutionary dynamics focusing on reciprocal interactions between ecology and evolution [34]. To explore key factors governing such a complex process in pathogenic bacteria, the adaptation process of *P. aeruginosa* PAO1 in a spatiotemporally heterogeneous environment was evaluated in this study by implementing an experimental evolution approach. As a result, two core evolutionary trajectories of PAO1 were described phenotypically and genomically; the one is the emergence and dominance of the hyperbiofilm variants, and the other is the rapid proliferation of the pili-defective variants having phage resistance.

The parallel evolution of hyperbiofilm wrinkly variants in our experiments indicates a strong positive selection for enhanced sessile growth modes (Figure 1). Emergence of hyperbiofilm variants was not observed in the evolution experiment under spatially-homogeneous conditions (Supplementary Figure S2), strongly indicating that spatial structure of habitat crucially contributed to adaptive evolution of hyperbiofilm phenotype. Biofilm formation at the air–liquid interfaces confer a fitness advantage by enabling cells to acquire oxygen more efficiently than cells in the broth phase, which has been demonstrated in some aerobic bacteria such as *Pseudomonas fluorescens* [31] and *Bacillus subtilis* [35]. Thus, once hyperbiofilm variants emerged, they swept out other types of variant in most cases due to their competitive phenotype. Similar results were also observed in an experimental evolution system with *Burkholderia* sp. [36], which described the evolution of hyperbiofilm lines in a population of biofilm formed at the solid surface. Although in vivo roles of enhanced biofilm formation ability might be habitat- and species-dependent and should be evaluated specifically to each environment, these previous and our studies support that the evolution of hyperbiofilm phenotype is a core evolutionary trajectory widely employed by sessile-living bacteria in various structured environments. The genomes of hyperbiofilm variants (Dm-1, F-1, CW-1, W-1, and W-2; Table 1) harbored mutations in *wspA* (PA3708), *wspF* (PA3703), and *yfiB* (PA1119) genes related to the production and degradation of c-di-GMP which functions as an intracellular second messenger regulating various cellular processes including exopolysaccharides production and motilities [37]. PA0171 and *morA* are also known as genes related to c-di-GMP metabolism [38,39], and mutations in these genes likely resulted in a moderate increase of biofilm formation (Um-1, Um-2, Sm-1, and Sm-2). Though only the CW-2 hyperbiofilm variant did not have mutations in any coding regions of its genome, a mutation was detected in a flanking upstream region of *siaA* (PA0172), which is a gene related to c-di-GMP metabolism and has been shown to be involved in cell aggregation [40]. These results suggest that the hyperbiofilm phenotypes emerged in our experiments resulted from the modulation of c-di-GMP signaling pathway (Figure 2). Given that there are many genes related to c-di-GMP metabolism in the genome of *P. aeruginosa* (>40 in the PAO1 genome [41]), there would be a large evolutionary opportunity to generate hyperbiofilm phenotypes by spontaneous mutation.

In contrast to hyperbiofilm variants, the emergence of smooth variants such as Sm-1 and Sm-2 was observed in a limited number of culture lines, and they rapidly persisted in the population (Figure 1). Although an increase of biofilm formation at the air–liquid interfaces was visually observed in the culture lines predominated by hyperbiofilm variants, biofilm amount and turbidity of culture broth apparently decreased when smooth variants were dominant in the population. Our results suggested that the proliferation of smooth variants was driven by a selective sweep of phage-susceptible variants, and this was consistent with the results by Davies et al., which demonstrated that external addition of temperate phages increased the frequency of twitching motility-deficient variants in successive cultures with artificial sputum medium [42]. The loss of type IV pili was observed in many clinical isolates obtained from patients with long-term chronic CF [43,44], and it has been explained by fitness advantage in vivo by avoiding phagocytosis by neutrophils [45]. Apart from this relationship with the host immune system, the present results support that the loss of type IV pili can be selected also by escaping phage infection. It should be noted that, while type IV pili are renowned for their key roles in biofilm formation (e.g., surface attachment and migration [46]), expression of type IV pili can be an unfavorable trait in terms of phage susceptibility and thus be maladaptive in the process of survival and adaptation even under biofilm-forming conditions. 

Population structure of *P. aeruginosa* in most culture lines transited from Um dominance to proliferation of hyperbiofilm or smooth variants, irrespective of the pure culture or the cocultures (Figure 1b and Figure 4). Furthermore, the transition of diversity index within each culture line showed a similar overshooting trend among culture series (Supplementary Figure S4; initial rapid diversification followed by gradual decrease), especially in the 1st experiment, and the PM series in the 2nd experiment exhibited fluctuating dynamics. These fluctuating dynamics can be explained by low diversity in some replicates (PM-7, PM-9, and PM-10) at mid time points (no. [day] 7 to 12). It was resulted from sustained Um dominance caused by delayed emergence of hyperbiofilm or smooth variants (Figure 1b), and the evolutionary path followed was not largely different from those in other experimental series (Figure 4). Thus, *S. aureus* strain Mu50 seemed to alter the rate of adaptive evolution of *P. aeruginosa*, though it was observed only in one experiment so that further trials and analysis are needed to elucidate this point. Overall, the difference of *P. aeruginosa* evolutionary trajectories between the pure culture and the coculture lines was marginal in the present study (Figure 4), indicating that interactions with *S. aureus* less affected the *P. aeruginosa* evolution than the spatial heterogeneity. Although the proliferation of the Sm variants at relatively early time points was uniquely observed in the coculture series with *S. aureus* strains compared with the pure culture series (e.g., PN-10 and PM-6 in Figure 1b and Figure 4), it is not evident at present that coexisting *S. aureus* strains are involved in the emergence of the Sm variants. Since the phage lysis in biofilm cells is induced by stressors such as reactive oxygen species [47], the competing *S. aureus* strains could impose the stress factors on *P. aeruginosa* cells. In fact, a work by Tognon et al., which performed transcriptomic profiling of *P. aeruginosa* and *S. aureus* in the co-culture of them, has revealed that *S. aureus* can induce stress responses of *P. aeruginosa* [22]. Another work performed by the same group has experimentally demonstrated that repetitive interactions with *S. aureus* Newman led *P. aeruginosa* PA14 to evolve into lipopolysaccharide (LPS)-deficient mutants [22], although the mechanistic link between the LPS loss and fitness advantage in the coculture with *S. aureus* has not been explored yet. We have not detected any evolved variants harboring mutations in LPS-related genes, but it is likely attributed to the difference in strains used and in the strength of interspecies interactions; they kept *S. aureus* high density in the coculture by externally adding considerable amount of *S. aureus* cells at every transfer, while we followed spontaneous population dynamics of coculture resulting in smaller population size of *S. aureus* (Figure 1b). Based on these recent works on interactions and evolutionary consequences of *S. aureus* and *P. aeruginosa* and our present study, the role of eco-evolutionary dynamics in the adaptation process of both pathogens is highly context-dependent (e.g., extent of spatial heterogeneity, strength of interspecies interactions). 

Evolutionary dynamics of PAO1 in the present experiments exhibited a typical pattern of clonal interference. It should be noted that neutral mutations and synonymous nucleotide variants were rarely found in the genome of CMVs in the present study. This likely indicates that the relevant CMVs rapidly predominated before such neutral nucleotide variants occurred and persisted in populations. The increase of biofilm formation and the phage resistance in *P. aeruginosa* posed strong positive selection, allowing these two processes to govern the observed evolutionary dynamics, and these two processes are not mutually exclusive (e.g., W-2 harbors mutations in both c-di-GMP-related and pili-related genes). Although the increase of biofilm formation would constantly confer strong fitness effects [36] and thus can be stably maintained in the local population, the fitness effect of phage resistance by the pili-deficiency would be conditional so that persistence of this phenotype is transitive; it would emerge and disappear repeatedly in the course of adaptation. Indeed, after the complete dominance of Sm, non-Sm variants emerged and dominated in some cases (e.g., line PN-10 and PM-6, Figure 1b), and such non-Sm variants included phage-susceptible variants (Supplementary Figure S5). The genetic backgrounds of these phage-susceptible non-Sm variants are not clarified yet but such variants are possibly pili-positive revertants originated from the Sm variants. Since a defect of pili resulted in a fitness decrease in phage-free environments (data not shown), compensatory mutations conferring fitness gain would be selected after phage concentration decreased enough. Thus, these two core processes (evolution of the hyperbiofilm variants and the pili-defective variants) have distinct characteristics in terms of fixation patterns in the evolving population. 

In addition to the two core evolutionary processes, the emergence and the proliferation of Um variants might be a key evolutionary trajectory as well because it was observed in all culture lines (Figure 1b and Figure 4). Since the Um variants often emerged prior to or parallel to other CMVs, the Um variants are likely in transitive genomic and/or phenotypic states between ancestor and the hyperbiofilm variants during the adaptation process. While the genomes of Um variants isolated from later time points (Um-1 and Um-2) harbored some mutations, the Um clone (Um-3) isolated from mid time point (7th transfer) possessed no mutations in the genome so that genetic features characterizing the Um phenotype (the morphology and the slight increase of biofilm formation) are unclear. Further genetic and phenotypic characterization of the Um variants will lead to a deeper understanding of the adaptation process of *P. aeruginosa* in heterogeneous environments.

## 5. Conclusions

The observed evolutionary dynamics of PAO1 provides important insights into the adaptive divergent process of *P. aeruginosa* in spatially heterogeneous environments; the variable diversification pattern was mainly governed by the two core trajectories shaped by extrinsic and intrinsic factors, the spatial heterogeneity of habitat which drives the biofilm growth mode, and the lysis by temperate phage which selects for the pili-deficiency, respectively. The interaction with *S. aureus* can potentially be an extrinsic factor playing important roles in the complex adaptation process of *P. aeruginosa* population in multispecies relationships, but it might be dependent on the strength of the interaction. Given that phage therapy draws much recent attention as an alternative or a complement of antibiotic treatment [48,49], prediction of the emergence of resistance to specific phage is very important. The present result implies that it should be careful about the rapid emergence of resistance when phages targeting type IV pili as binding site are used against *P. aeruginosa*.

## Figures and Tables

**Table 1:** Nucleotide variants in the genomes of isolated CMVs.

CMV (Colony Morphology)	Origin ^1^	Chromosomal Position, Type of Variation, Amino Acid Change	Gene	Predicted Function
Um-1 (Umbonate)	P-1-21	194365, In frame deletion (4 aa)	PA0171	c-di-GMP metabolism
Um-2 (Umbonate)	P-4-21	5159267, SNV ^2^, Arg967→Leu	*morA* (PA4601)	c-di-GMP metabolism
Um-3 (Umbonate)	PN-1-7	N.D. ^3^	-	-
Dm-1 (Dome)	P-3-19	4145826, SNV, Gln41 *	*wspF* (PA3703)	c-di-GMP metabolism
F-1 (Fried-egg)	PN-3-13	4151113, SNV, Ala411→Val	*wspA* (PA3708)	c-di-GMP metabolism
CW-1 (Center-wrinkly)	P-1-21	1213097, SNV, Leu43→Phe	*yfiB* (PA1119)	c-di-GMP metabolism
CW-2 (Center-wrinkly)	PM-3-21	196821, SNV	Intergenic region	Upstream region of *siaA* (PA0172)
W-1 (Wrinkly)	P-1-21	4151032, SNV, Ala438→Val	*wspA* (PA3708)	c-di-GMP metabolism
W-2 (Wrinkly)	PN-1-21	4151496, SNV, none 5069263, SNV, Gly90→Asp	*wspA* (PA3708) *pilA* (PA4525)	c-di-GMP metabolism Type IV pilus protein
Sm-1 (Smooth)	PN-1-21	5069340, SNV, Ser64→Arg 194365, In frame deletion (4 aa)	*pilA* (PA4525) PA0171	Type IV pilus protein c-di-GMP metabolism
Sm-2 (Smooth)	PN-2-21	452798, SNV, Ala557→Thr 5159824, SNV, Glu1153→Lys	*pilJ* (PA0411) *morA* (PA4601)	Twitching motility related gene c-di-GMP metabolism

^1^, Series-Line-No. of transfers correspond to Figure 1b; ^2^, Single nucleotide variants; ^3^, Not detected; *, nonsense codon.

## Data Availability

Sequence data of genome resequencing analysis for evolved variants have been submitted to the DDBJ/EMBL/GenBank databases under the accession number DRA007357 (https://ddbj.nig.ac.jp/DRASearch/submission?acc=DRA007357 (accessed on 8 February 2021)).

## Supplementary Materials

The following are available online at https://www.mdpi.com/2076-2607/9/3/569/s1, Figure S1: Complete data sets for transition of population size and abundance of CMVs, Figure S2: Transition of colony morphology of *P. aeruginosa* in the course of evolution experiment under spatially-homogeneous conditions, Figure S3: Detection of replication-form DNA of Pf4, Figure S4: Transition of diversity, Figure S5: Phenotypic features of non-smooth CMVs predominated after Sm dominance.
