# Supplementary material for: Parallel Evolution of Enhanced Biofilm Formation and Phage-Resistance in Pseudomonas aeruginosa during Adaptation Process in Spatially Heterogeneous Environments
Source: Microorganisms. 2021 Mar 10;9(3):569. doi: 10.3390/microorganisms9030569 (PMC7999436; doi:10.3390/microorganisms9030569)
Supplement: Supplementary file 1 [file microorganisms-09-00569-s001.pdf]

Supplementary Material

Parallel evolution of enhanced biofilm formation and phage-resistance in *Pseudomonas aeruginosa* during adaptation process in spatially heterogeneous environments

Kyosuke Yamamoto[1,2,3*], Hiroyuki Kusada[2], Yoichi Kamagata[2], Hideyuki Tamaki[2,3,4*]

[1] Bioproduction Research Institute, National Institute of Advanced Industrial Science and Technology (AIST), Sapporo, Hokkaido, Japan.

[2] Bioproduction Research Institute, AIST, Tsukuba, Ibaraki, Japan.

[3] Faculty of Life and Environmental Sciences, University of Tsukuba, Tsukuba, Ibaraki, Japan.

[4] Biotechnology Research Center, University of Tokyo, Bunkyo-ku, Tokyo, Japan.

***Correspondence**

Kyosuke Yamamoto (k.yamamoto@aist.go.jp) and Hideyuki Tamaki (tamaki-hideyuki@aist.go.jp)

Supplementary Figure S1 (Yamamoto et al.)

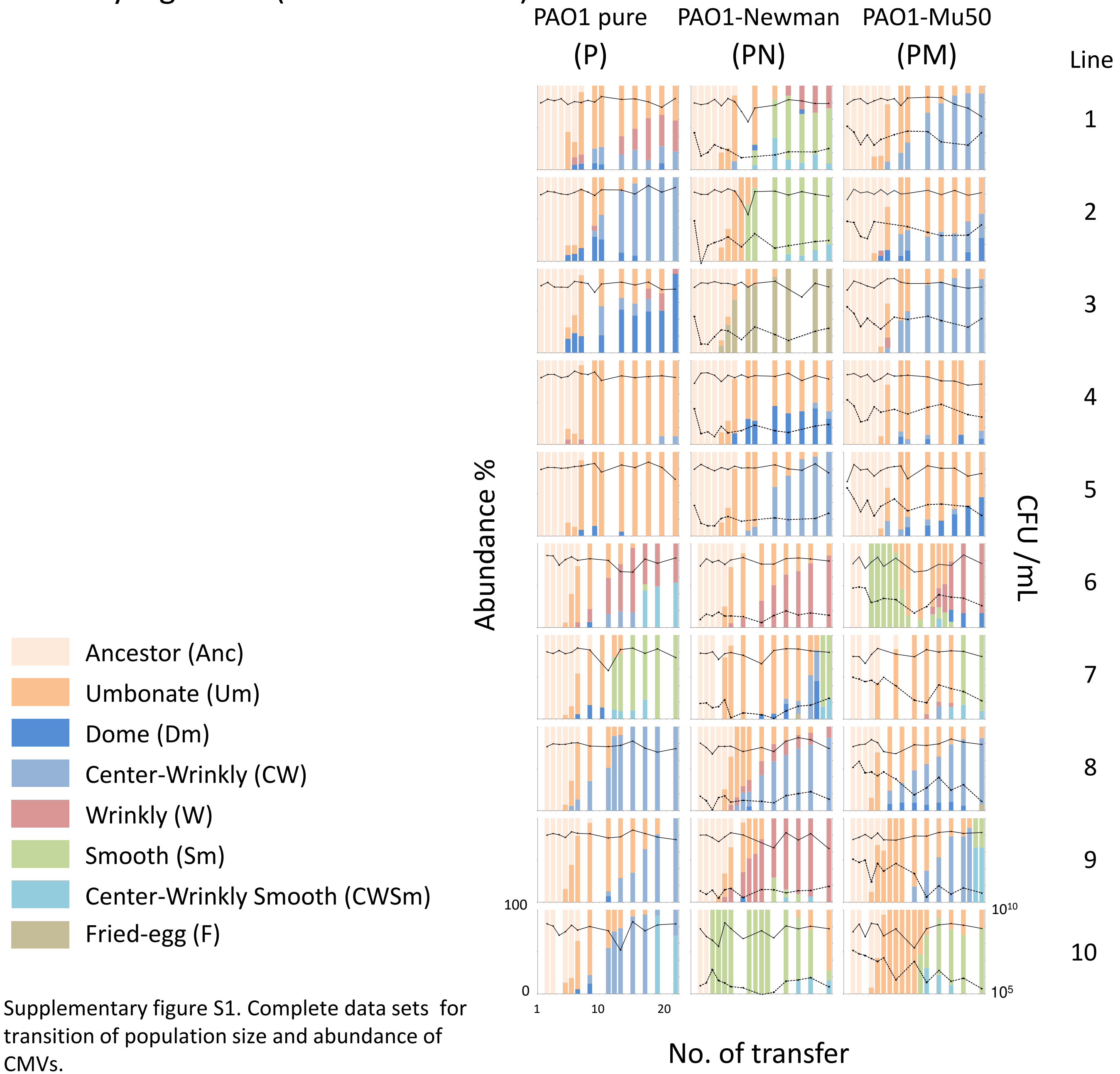

Supplementary figure S1. Complete data sets for transition of population size and abundance of CMVs.

Supplementary Figure S2 (Yamamoto et al.)

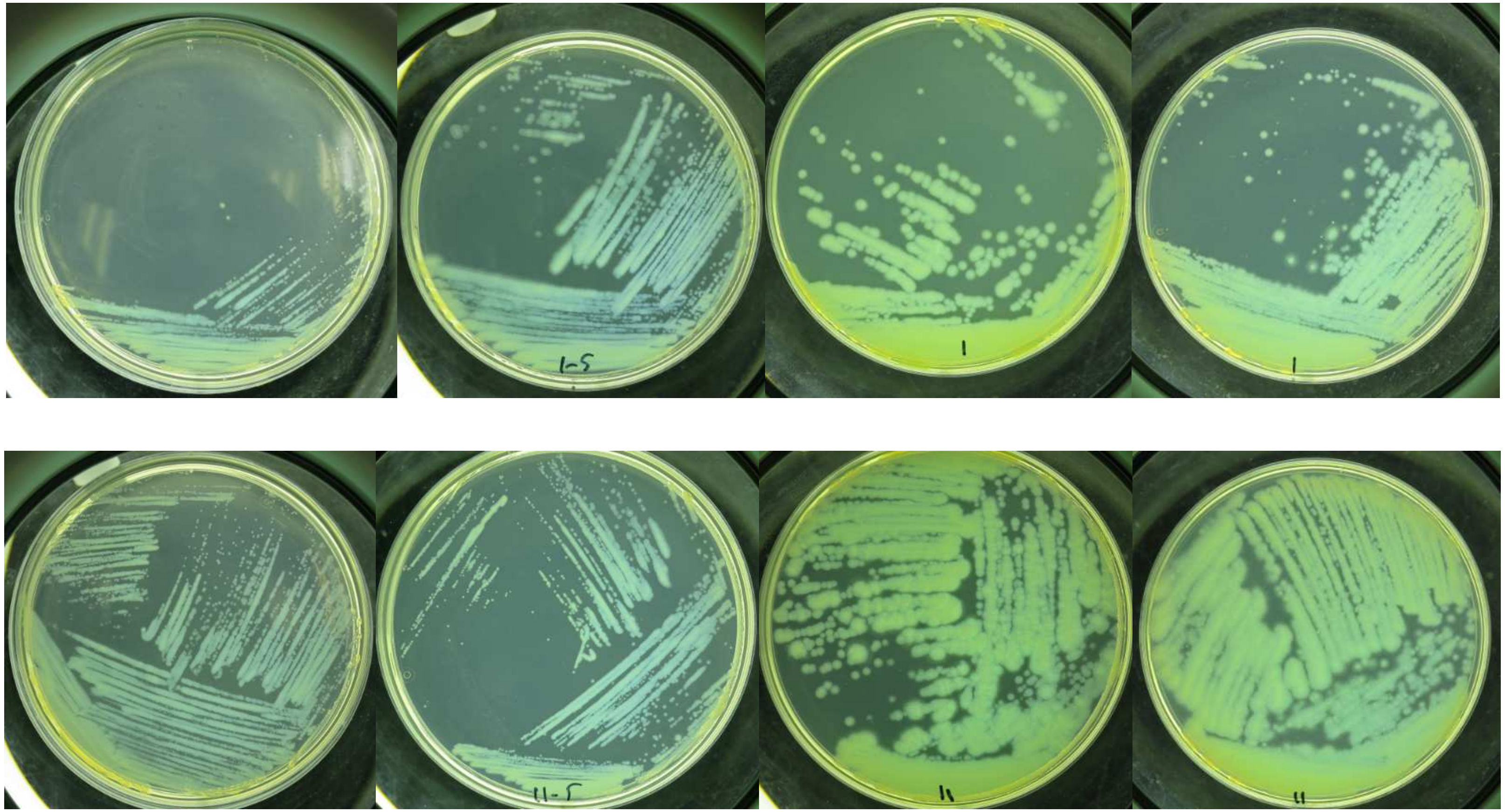

Supplementary figure S2. Transition of colony morphology of PA in the course of evolution experiment under spatially-homogeneous conditions. The evolution experiment of PA pure culture was conducted in shaken test tube using same culture conditions as the experiment under static conditions except for continuous agitation (200 rpm). The culture broth at 1st, 5th, 10th, and 15th round of passages were streaked on TSB plates and then incubated at 30°C. Colony morphology was checked after 18~36h incubation. N = 20. Colony of replicate No. 1 and 11 are shown as representatives.

Supplementary Figure S3 (Yamamoto et al.)

a

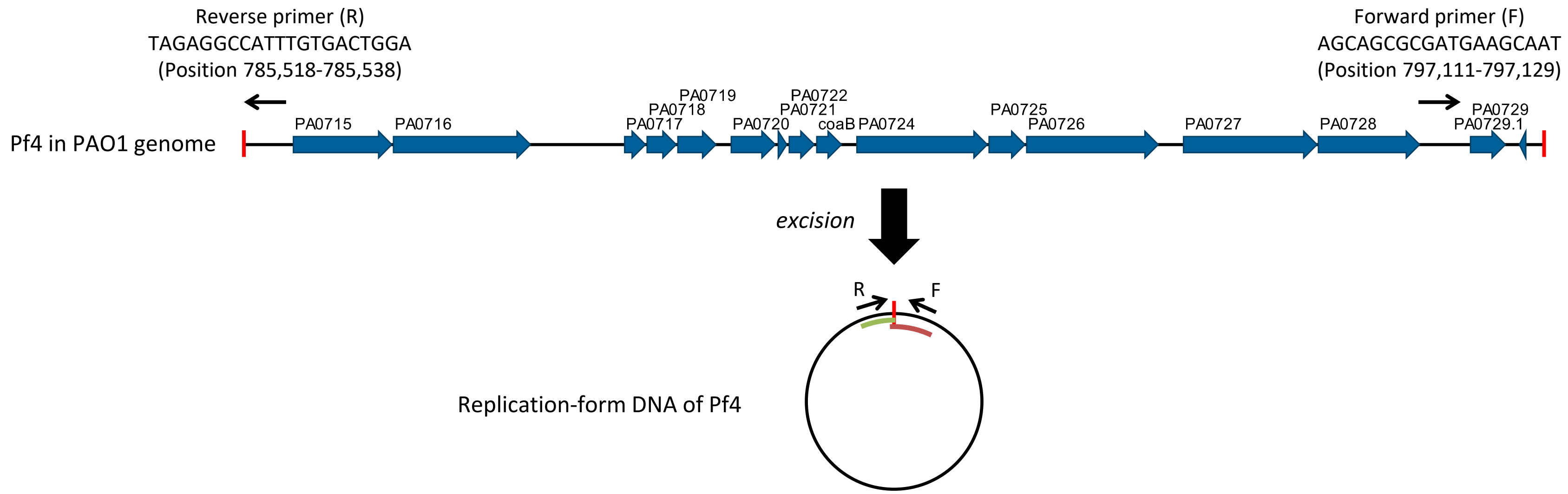

b

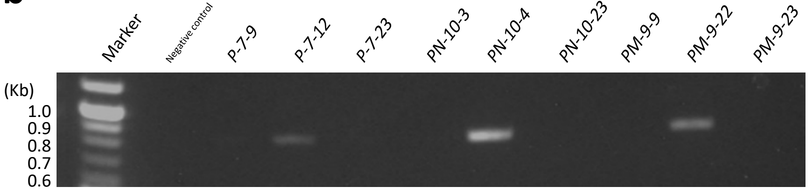

c

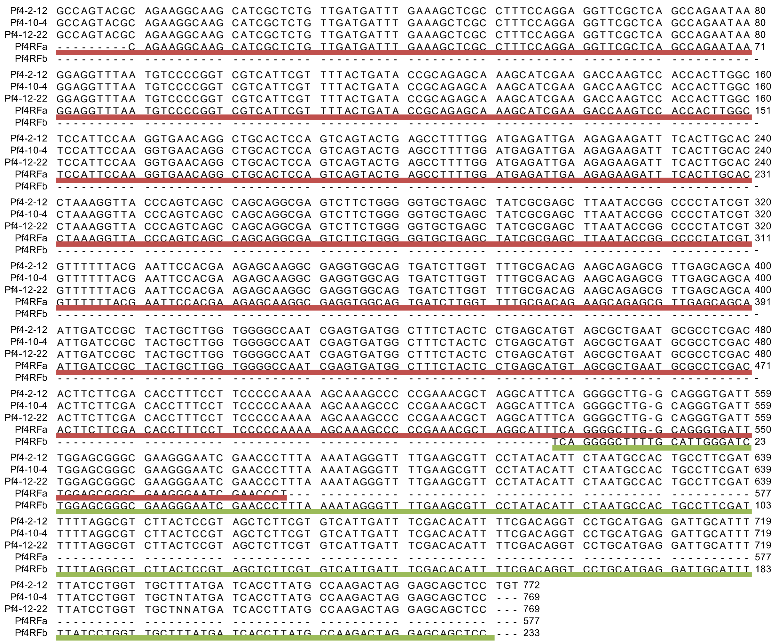

Supplementary figure S3. Detection of replication-form DNA of Pf4. **a**) Position of Pf4 in the PAO1 genome. Green and brown bars indicate regions used for sequence alignment. **b**) PCR analysis was performed with primer sets (arrows in a) specifically amplifying replication-form DNA of Pf4: forward (5'-AGCAGCGCGATGAAGCAAT-3') & reverse (5'-TAGAGGCCATTTGTGACTGGA-3') (Webb et al., 2004. J. Bacteriol. 186, 8066–8073). The expected PCR product size was 839-bp. Templates were the heat-treated culture filtrates (0.22um; 100˚C, 15 min). **c**) Sequence alignment of the PCR products and Pf4 region of PAO1 genome. Pf4-2-12, Pf4-10-4, and Pf4-12-22 corresponds to P-7-12, PN-10-4, and PM-9-22 in b, respectively. Pf4RFa and PF4RFb correspond to the regions indicated in a. Sequencing primer for the PCR products was the forward primer used for PCR amplification. Sequence alignment was performed with CLC Main Workbench (Qiagen).

Supplementary Figure S4 (Yamamoto et al.)

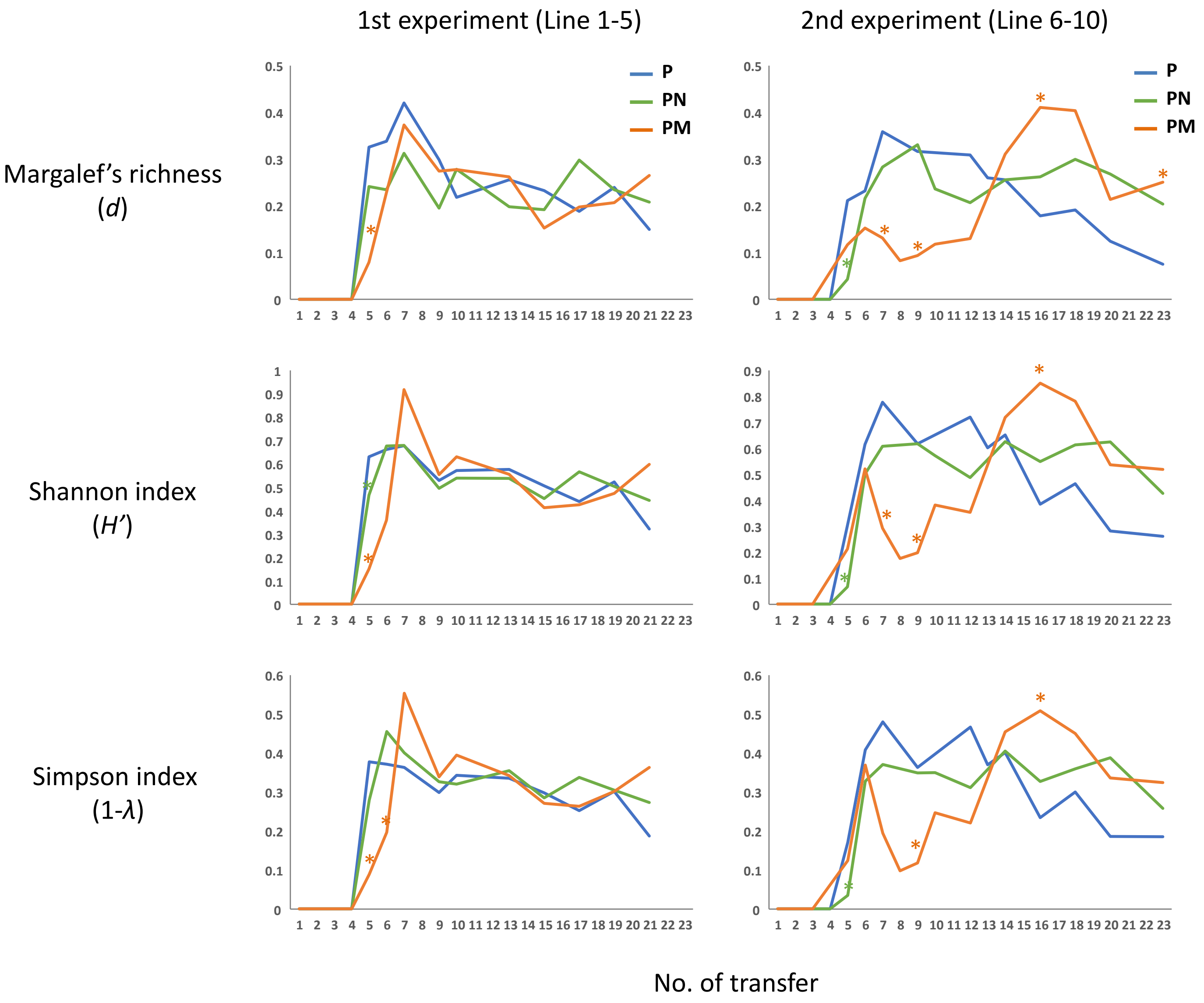

Supplementary figure S4. Transition of diversity. Three diversity index (Margalef's species richness, Shannon's index, and Simpson's index) were calculated in all time points using population structure data. Transition of diversity was separately presented by two independent experiments. Margalef's species richness: *d* = (S-1)/Log(N); S, the number of species in each sample; N, the number of individuals in each sample. Shannon's index: H'= SUM(p_i*Log_e(p_i)); p_i, the proportion of individuals belonging to the *i*th species in the dataset. Simpson's index: 1-λ = 1-SUM(p_i^2). Values were median of 3-5 replicates. Calculation was performed with PRIMER 6 software. * time point having statistically significant difference (t-test, p<0.05) with the pure culture lines (P). There was no time point having statistically significant difference among all three culture series (ANOVA, p<0.05).

Supplementary Figure S5 (Yamamoto et al.)

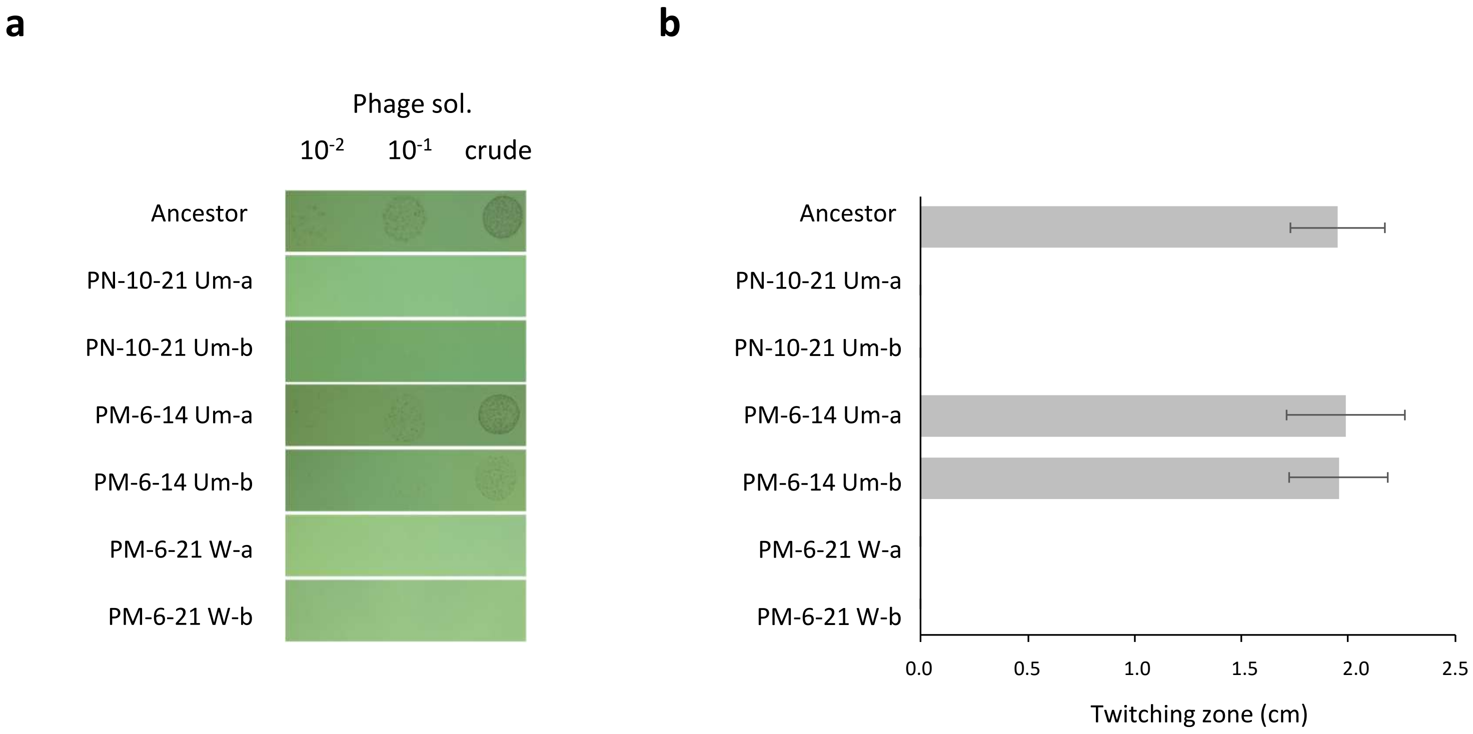

Supplementary figure S5. Phenotypic features of non-smooth CMVs predominated after Sm dominance. **a**) Susceptibility to temperate phage. Phage solution and its dilution series were spotted onto a lawn of strains indicated. **b**) Twitching motility (48 h). Values are expressed as means for two independent experiments (n=3-6). Error bars indicate SD. N.D., Not detected.
